# Parametric Study of Gold Nanoparticles Synthesis under Micro-Continuous Flow Conditions

**DOI:** 10.3390/molecules27248651

**Published:** 2022-12-07

**Authors:** Mohannad T. Aljarrah, Ala’a M. Alboull, Mohammad S. Alharahsheh, Azad Ashraf, Amith Khandakar

**Affiliations:** 1 Department of Chemical Engineering, College of Engineering, Jordan University of Science and Technology, Irbid 22110, Jordan; 2 Department of Chemical Engineering, College of Engineering Technology, University of Doha for Science and Technology, College of Engineering Technology, Doha P.O. Box 24449, Qatar; 3 Department of Electrical Engineering, College of Engineering, Qatar University, Doha P.O. Box 2713, Qatar

**Keywords:** gold nanoparticles, micro-reactors, flow synthesis

## Abstract

The synthesis of gold nanoparticles (GNPs) using chemical reduction in batch and microreactor methods has been reported. A parametric study of the effect of several parameters on the size of gold nanoparticles was performed in batch synthesis mode using the modified Martin method. The best-obtained conditions were used to synthesize gold nanoparticles using a glass chip microreactor, and the size of the resulting GNPs from both methods was compared. The presence of polyvinyl alcohol (SC) was used as a capping agent, and sodium borohydride (SB) was used as a reducing agent. Several parameters were studied, including HAuCl_4_, SC, SB concentrations, the volumetric ratio of SB to gold precursor, pH, temperature, and mixing speed. Various techniques were used to characterize the resulting nanoparticles, including Atomic Absorbance spectroscopy (AAS), Ultraviolet-visible spectroscopy (UV-Vis), and dynamic light scratching (DLS). Optimum conditions were obtained for the synthesis of gold nanoparticles. Under similar reaction conditions, the microreactor consistently produced smaller nanoparticles in the range of 10.42–11.31 nm with a reaction time of less than 1 min.

## 1. Introduction

Gold nanoparticles (GNPs) are extremely small particles ranging in size from one nanometer to one hundred nanometers [1]. Because of their many distinguishing physical and chemical properties [2,3], non-toxicity, low cost, and ease of use, the world has turned to the use of GNPs in many fields. GNPs have been used in a variety of applications, including drug delivery [4,5], catalysis [6], and medicine [7]. Many variables influence GNP synthesis, including pH [7], temperature [8], reducing agent [9] and gold precursor concentrations [10], mixing speed [11], synthesis time [12], and volume or molar ratio of (stabilizing or reducing) agent/HAuCl_4_ [8,13]. 

Many reduction agents, including sodium citrate (SC) [14,15], sodium borohydride (SB) [16,17,18], ascorbic acid (AscH_2_) [19,20,21], and hydrazine [22,23], have been used to synthesize gold nanoparticles. Faraday [24] synthesized colloidal GNPs for the first time in 1857 by reducing them with phosphorous. Brust [25] created GNPs in 1871 by reducing HAuCl_4_ with SB in an organic liquid that was not miscible with water, such as toluene. The gold is then transferred to the toluene phase using the transmission factor in the presence of a capping agent, Dodecane-thiol. In 1951, the Turkevish method used citrate as a reducing and capping agent to synthesize GNPs [26]. One of the biggest limitations of this method is its inability to use water as a solvent. Sodium citrate has important advantages over other reduction agents, such as ascorbic acid. In addition to being environmentally friendly, sodium citrate provides both monodispersed and spherical shapes for GNPs. Perrault [27] produced GNPs in 2009 using hydroquinone as a reducing agent. The reduction of HAuCl_4_ in an aqua solution containing GNP seeds is the basis for this technique. The Martin method [28] was used in 2010 to produce naked GNPs by reducing HAuCl_4_ with SB without the use of any stabilizer or capping agent. Sodium borohydride is one of the strongest reducing agents, but it produces a lot of unstable nanoparticles [29].

One of the most difficult challenges researchers faced when synthesizing nanoparticles (NPs) in batch methods was their inability to achieve consistent results and efficiency from batch to batch [30]. Another issue with batch mode synthesis is inhomogeneous size to nanoparticle instability [31]. As a result, the researchers sought alternative methods and solutions for synthesizing GNPs with uniform properties. Because of their high mixing efficiency, microreactors have grown in popularity. Microreactor technology has recently replaced batch methods as the primary method for synthesizing NPs owing to its numerous advantages [24]. The batch method requires a long residence time, and because of its large dimensions, it takes longer than a microreactor with small dimensions that stimulates interaction.

Microreactors are techniques for creating chemical reactions in very small dimensions of less than a millimeter [32]. Because of the microsize and the very high surface area to volume ratio [33,34], which varies in the range of (10,000–50,000 m^2^/m^3^) [12], the use of microreactors results in good mixing. As a result, the NP size distribution improves. Furthermore, microfluidic devices have many features that have influenced the world’s preference for using them in a variety of applications. One of the most significant advantages of microfluidic devices is the ability to precisely control the shape and size of the resulting gold nanoparticles. The flow within the reactor divides the microreactor into two types: single-phase (continuous flow) and multi-phase flow (segmented flow) [35].

Continuous flow is the most commonly used in the reactions, but the biggest disadvantage of this type is the adhesion of GNPs formed on the surface of the wall reactor due to the influence of the boundary layer. The boundary layer effect arises from the laminar flow regime commonly found in microreactors, which causes the well-known parabolic fluid velocity distribution. The velocity distribution can cause uneven mixing in the axial direction and hence affect the residence time. Different residence times inside the reactor affect the mono-dispersity of the nanoparticles. Another issue is associated with the contact of nanoparticles with the reactor wall due to the high surface energy of the nanoparticles and the low fluid velocity near the wall, which causes the particles to stick to the wall casing clogging of the microreactor [1]. For the first time, ascorbic acid was used as a reducing agent mixed with gold seeds of 12 nm to synthesize GNPs using a microreactor as per Wagner et al. [36]. GNPs with a size of 24 nm were achieved. In 2005, Wagner et al. [37] used the microreactor for the second time. GNPs were produced using ascorbic acid as a reduction agent in the absence of gold seeds and using PVP as a capping agent to raise the pH value, preventing the adhesion of GNPs formed to the surface of the reactor. In 2005, Köhleret al. [38] used a microreactor consisting of three micromixers. In this reactor, ascorbic acid and iron (II) sulfate were used as reducing agents in the presence of polyvinyl alcohol as a capping agent. In addition, sodium metasilicate was used to facilitate the clustering of GNPs. Wagner et al. [39] used the microreactor for the third time in 2008, obtaining GNPs of 3–5 nanometers when sodium was used as a reduction agent. In 2008, Tsunoyama et al. [40] investigated the PVP: Au molar ratio in the presence of sodium borohydride as a reducing agent. Luty-Bocho et al. [41] investigated the effect of flowrate from (7.5 to 0.1) mL/min on the final size of GNPs in a microreactor in 2011. They used ascorbic acid as a reducing agent in the presence of PVP as a stabilizer to reduce gold precursors. GNP sizes ranging from 1.7 to 33 nm were obtained. The pH effect on the size of GNP was investigated using a Teflon mixer connected to a capillary tube by Ishizaka et al. [42]. They used glucose as a reducing agent. In 2012, Ftouni et al. [43] was able to synthesize monodispersed GNPs with sizes less than 2 nm by controlling synthesis parameters in a microreactor using conventional Turkevich with SC/HAuCl_4_**.** In this procedure, the gold precursor is reduced with sodium citrate at its boiling point. The impact of the residence time and temperatures was investigated using a Y-mixer/PTFE microreactor at a constant temperature range of 298–333 K by Sugie et al. [44]. In this work, they used organosilanes (RSiH) as a reducing agent to synthesize homogenous GNPs in the presence of an alkyl thiol (RSH).

To overcome the problem of GNP adhesion to the reactor wall caused by the boundary layer effect in continuous flow, some scientists used a flow called segmented flow [45]. This type of flow is distinguished by the presence of a transporter medium, which is frequently alcohol, air, or oil. Khan and Duraiswamy [46] were the first to create GNPs by SB using an inert gas with segmented flow within the microreactor. The inert gas was intended to absorb and reduce the solubility of hydrogen gas inside the reactor. Sebastian Cabeza [47] created GNPs by reducing them with SB using the inert fluids toluene and silicon oil in the microreactor’s segmented flow.

The aim of this work is to conduct a comprehensive parametric study on the synthesis of GNPs under microfluidic conditions using the modified Martin method using sodium citrate and polyvinyl alcohol. The quality of the synthesized GNP is then evaluated using atomic absorption spectroscopy (AAS), ultraviolet-visible spectroscopy (UV-Vis), dynamic light scratching (DLS), and Fourier-transform infrared spectroscopy (FTIR). The paper is divided into four parts. Section 1 discusses the problem statement and contributions of the paper, while Section 2 discusses the findings and offers some commentary on them, discusses the findings and offers some commentary on them. Section 3 discusses the methodology used in the paper. Finally, Section 4 provides the paper’s detailed investigation conclusion.

## 2. Results and Decision

This section presents the detailed investigation conducted in the paper. The section describes the effect of different parameters on the synthesized GNPs using the method proposed in the paper.

### 2.1. The Effect of the Best Conditions on the Particle Size

**Figure 1:**
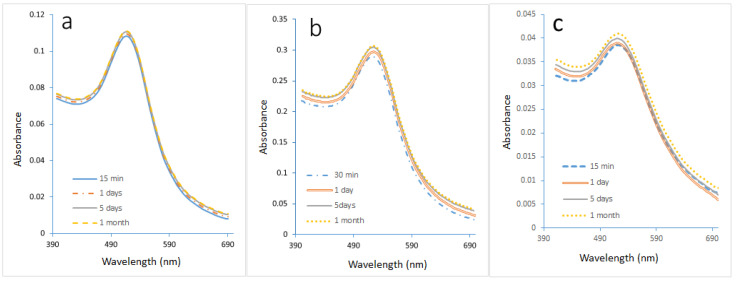
The UV-spectra of GNP samples prepared under optimal conditions in Microreactor mode. (**a**) in the presence of PVA stabilizer. (**b**) in the absence of the stabilizer. (**c**) In the absence of stabilizer and using the batch method.

The experimental conditions for the synthesis of GNPs in microreactors were chosen based on the best results obtained in batch synthesis experiments. The optimal parameters for concentrations of HAuCl_4_, SC, and SB, the volumetric ratio of SB to gold precursor, pH, and temperature were obtained at 0.1 mM, 0.1 mM, and 0.1 mM, 4:10, 3, and 308.15 K, respectively. Figure 1 demonstrates the effects of the best conditions on particle size. The final size of GNPs is dependent on the maximum wavelength. Figure 1a shows that the UV spectra are almost identical with one narrow peak, indicating the presence of GNPs of uniform shape and size. Furthermore, the maximum wavelength of GNPs is constant, indicating that the constant size of GNPs has not changed. The maximum wavelength of GNP synthesis in the presence of PVA using a microreactor was 518.5 nm. Figure 1b shows that the reaction in the microreactor continued to grow, albeit very slightly, in the absence of the stabilizing agent. It can be seen that the maximum wavelength of the GNPs moved the peak from 520 nm to 522 nm, which indicates that the size of GNPs increased from 11.76 nm to 13.54 nm. UV spectra for GNP prepared using batch mode in the absence of PVA is shown in Figure 1c. The maximum wavelength of the GNP shifted from 522 nm to 528 nm, which implies that the size of GNPs increased from 17.37 nm to 26.76 nm.

**Figure 2:**
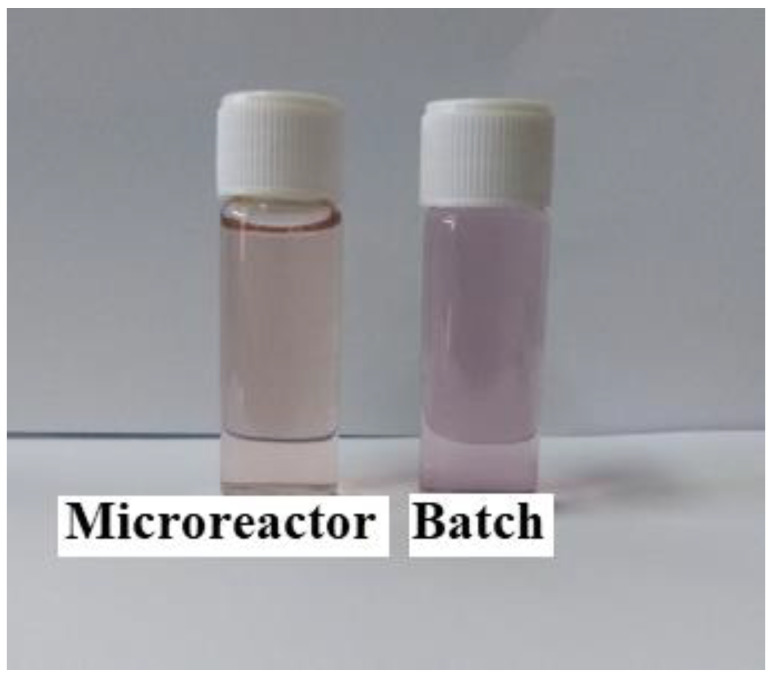
Colors of GNPs samples prepared under optimal conditions (Cons of SC, SB, HAuCl_4_ = 0.1 mM, initial pH = 3, and T = 308.15 K).

The size of GNPs is determined based on the UV chart. It was also observed how effective the microreactor was in obtaining small-sized GNPs compared to the batch (conventional) method, where GNPs with sizes ranging from 17.4 to 23.6 nm were obtained using the batch method. This was evident in the different colors of the solutions, as seen in Figure 2. In this study, the stock-gold solution mixed with SC solution was loaded into the first syringe and pumped at a rate of 5.55 × 10^−6^ L/s, while the SB solution in the other syringe was pumped at a high rate of 1.11 × 10^−5^ L/s. The flow rate of SB was greater than that of gold in order to ensure the saturation process, which helps the fast growth of gold nuclei. Furthermore, it proved to be the most effective method by using a high reactant flow rate to prevent the adhesion process due to decreasing the residence time in the reactor and increasing the mixing process. The residence time was calculated by this equation Ʈ = V/V°, where the volume of the microreactor was 92 µL, and the total flow rate was equal to 1.66 × 10^−5^ L/s. The residence time inside the microreactor was 5.52 s.

**Figure 3:**
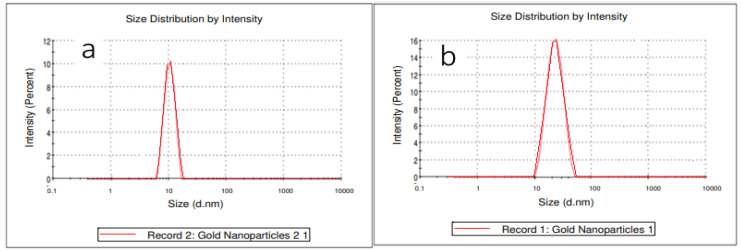
The frequency plot of GNPs prepared under optimal conditions in the presence of the stabilizer PVA. (**a**) using a microreactor, and (**b**) using a batch method (T = 308.15 K, total flowrate of SB and gold with SC = 1.66 × 10^−5^ L/s, volumetric ratio of SB/HAuCl_4_ = 4:10, pHi = 3, and concentration of HAuCl_4_, SC, and SB = 0.1 mM).

A DLS analysis was performed to verify UV-vis spectroscopy data about the effect of the best conditions on the size and size distribution of GNPs, as shown in Figure 3a,b. The figures show that the DLS size decreased when the GNP synthesis was performed in microreactor mode. The mean size of GNPs in this sample was 11.3 nm, whereas the mean size of GNPs in the batch mode sample was 25 nm. These DLS results are very close to the UV-Vis of this work, in which large GNPs were produced in batch mode, as shown in Figure 1a–c.

**Figure 4:**
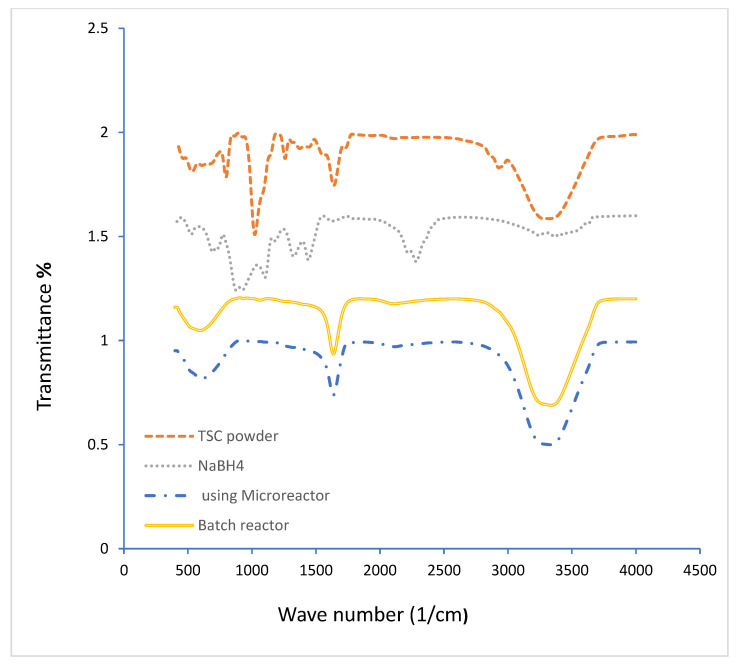
The FTIR spectra of GNPs prepared at various methods. (After 24 h, for batch and microreactor methods, T = 308.15 K, cons of SC, SB, HAuCl_4_ = 0.1 mM, 700 rpm, total flowrate of SB and gold with SC = 1.66 × 10^−5^ L/s, Volumetric ratio of SB/HAuCl_4_ = 4:10, pH_i_ = 3).

Figure 4 shows the GNPs’ FTIR spectra. GNPs’ citrate capping is structurally revealed by FTIR spectra. For comparison, the FTIR spectra of SB and TSC were used as a reference. It was determined that the strong peak at 3310 cm^−1^ is H-O. Also, a strong peak at 1630 cm^−1^ is identified as C=O due to the presence of TSC on the surface of GNPs in the form of a carboxyl group (COO-), where Tri-sodium citrate (Na_3_C_6_H_5_O) contains more than one group of (COO-). The gold solution’s low-wavenumber peak (595.15 to 602.85 cm^−1^) signified the metal’s binding to oxygen (Au-O). This indicates the complete coverage of sodium citrate on the surface of the GNPs.

### 2.2. The Effect of the Initial Gold Concentration

**Figure 5:**
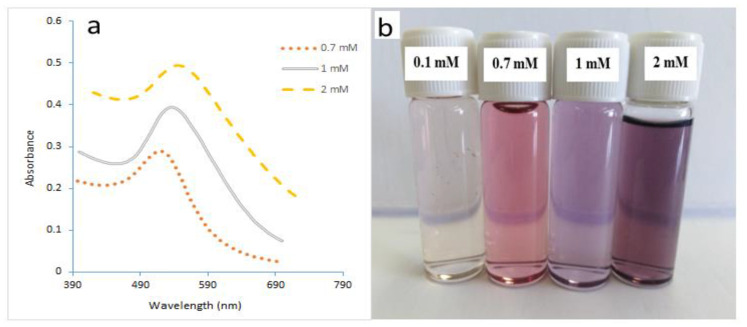
(**a**) UV spectra of GNPs samples prepared in a microreactor at various gold concentrations. (**b**) GNPs’ colors (Temperature = 308.15 K, total flowrate of SB and gold with SC = 1.66 × 10^−5^ L/s, Volumetric ratio of SB/HAuCl_4_ = 4:10, pH_i_ = 3).

Figure 5a shows the UV spectra of GNPs prepared at various initial HAuCl_4_ concentrations. It can be noted that low gold concentrations resulted in low absorption values, as well as a short wavelength of 516 nm. The absorption value increased as concentrations increased to 2 mM. It was also noticed that the peak of the curve had shifted from left to right. It can be seen that the wavelength shifted from 516 nm to 545 nm. The average particle size was (8.19–34.08) nm, with concentrations of HAuCl_4_ ranging from 0.1 to 2 mM. Figure 5b shows how the apparent color of the gold nanoparticle solution changes as the particle size changes. These results were similar to those achieved using the conventional method. The microreactor system did not change the batch Martin method’s reaction response pathway.

**Figure 6:**
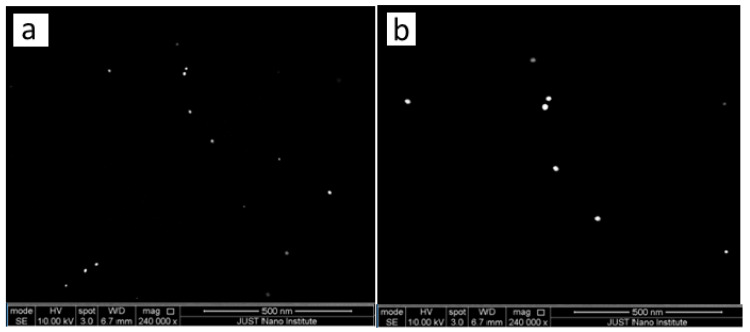
SEM images of the GNPs prepared at various HAuCl_4_ Concentration. (**a**) at 0.1 mM, (**b**) at 2 mM. (After one day. Temperature = 308.15 K, total flowrate of SB and gold with SC = 1.66 × 10^−5^ L/s, Volumetric ratio of SB/HAuCl_4_ = 4:10, pH_i_ = 3).

**Figure 7:**
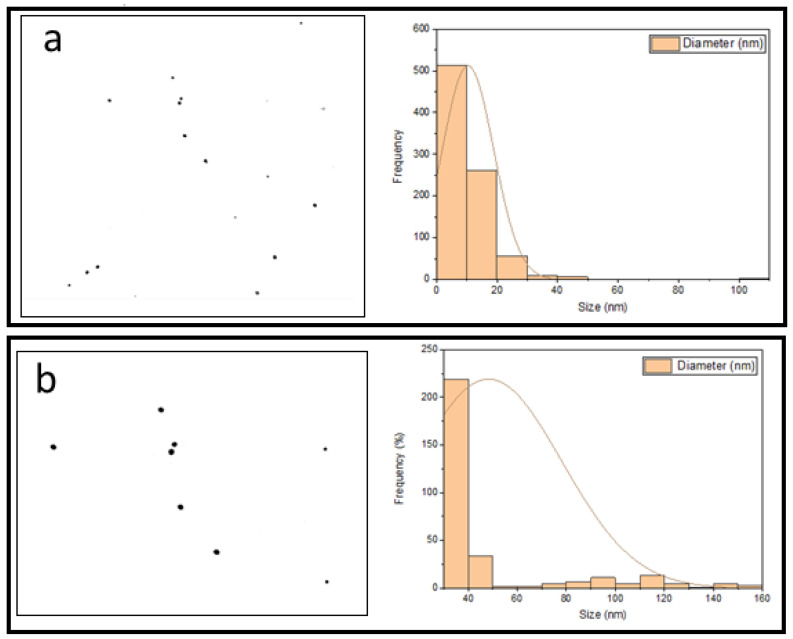
SEM images of the GNPs prepared at various HAuCl_4_ Concentration. (**a**) at 0.1 Mm (left hand side), (**b**) at 2 mM (left hand side), Histogram of percent frequency distribution of gold nanoparticles (right hand side).

The SEM image displayed in Figure 6 and Figure 7a verified that the pale pink solution contained gold nanoparticles with a spherical ultra-small size of 10 nm. The second image in Figure 6 and Figure 7b shows a gold nanoparticle with a large size with an increasing concentration of gold nanoparticles. It can be seen that the higher concentration of HAuCl_4_ leads to bigger particles and more agglomeration. This was accompanied by an increase in size when using high concentrations of HAuCl_4_, which is why agglomerates formed in a shorter time when compared to lower concentrations. These results are consistent with the UV-vis results presented in the previous section of this study

**Figure 8:**
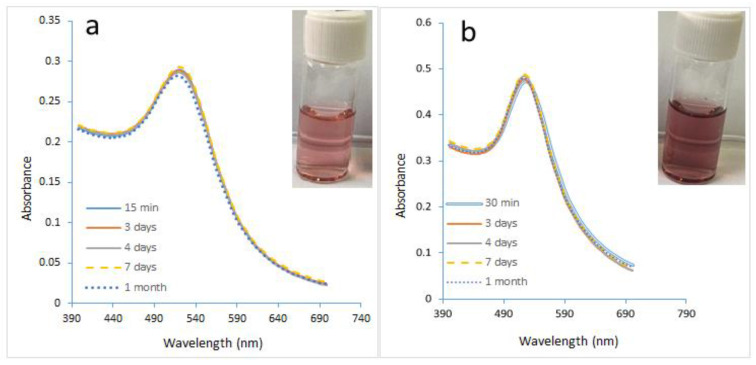
The UV spectra of GNPs samples prepared in the presence of PVA. (**a**) utilizing a microreactor, (**b**) batch method (Cons of HAuCl_4_ = 0.7 mM, Cons of SC, SB = 0.1 mM T = 308.15 K, Volume ratio of SB/HAuCl_4_ = 4:10, pH_i_ = 3).

**Figure 9:**
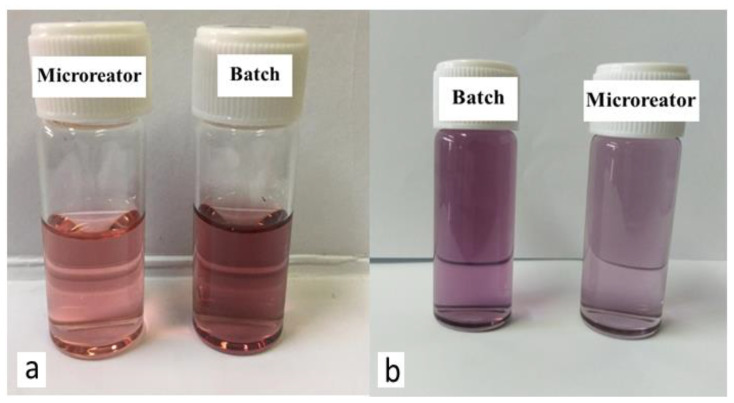
*(***a**) Colors for GNPs samples prepared in a microreactor with a HAuCl_4_ concentration of 0.7 mM. (**b**) with an initial HAuCl_4_ concentration of 2 mM.

Figure 8a,b show that highly stable GNPs were produced despite the high initial value of gold precursors of 0.7 mM. It was observed that GNPs prepared using the microreactor have a constant wavelength of 521 nm due to the use of a stabilizer. As to Figure 8b, it was noted that GNPs started with a slow growth process with a constant wavelength of 524 nm. The sizes of the GNPs prepared using a microreactor ranged in the presence of PVA from 15.8 to 18.2 nm, while the sizes of the GNPs prepared using a batch ranged in the presence of PVA from 20.5–23.63 nm. The size difference between the two samples was observed through their colors, as seen in Figure 9a.

**Figure 10:**
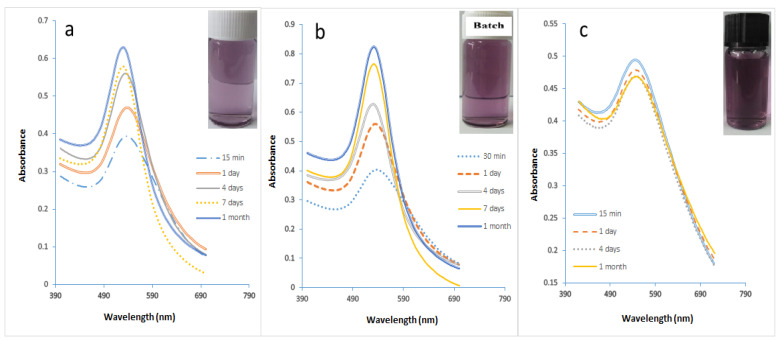
(**a**) The UV spectra of GNPs samples prepared in the presence of PVA in a microreactor at a concentration of HAuCl_4_ of 1 mM (**b**) using the batch method. (**c**) using a microreactor with an initial HAuCl4 concentration of 2 Mm. (Cons of SC, SB = 0.1 mM, T = 308.15 K, SB/HAuCl_4_ volumetric ratio = 4:10, pH_i_ = 3).

Figure 10a shows the UV spectra with narrower curves than the curves in Figure 10b. That confirmed the efficiency of the microreactor in producing the small size and uniform shape of GNPs. These results were confirmed by the various colors of the GNPs, as shown in Figure 9b. The sizes of GNPs prepared using a microreactor ranged in the presence of PVA from 39 to 42.4 nm, while the sizes of the GNPs prepared using a batch ranged in the presence of PVA from 48.66 to 58 nm.

These changes in peak absorption indicate the formation of larger GNPs. Furthermore, the broad trend of curves indicates dissimilar shapes and sizes of GNP. It can be observed that the absorbance peak (maximum wavelength) increased with increasing HAuCl_4_ concentration, indicating that high concentrations of GNPs were formed. The wavelength is directly related to the final size of the nanoparticles. It was found that the size of GNPs increased with the increasing concentration of HAuCl_4_, which is the reason behind enhancing the formation of agglomerates within a short time. The phenomenon of agglomeration is strongly dependent on the initial concentration of HAuCl_4_ and the number of GNPs, where the higher the concentration, the higher the surface energy of GNPs. Therefore, random movement and repeated collisions of GNPs enhance the adhesion process and the emergence of agglomeration. The increase in the number of nanoparticles in large numbers is another reason for the phenomenon of agglomeration associated with the increased concentrations of gold precursors. Nanoparticles become closer to each other, leaving them under the influence of the electromagnetic field of one particle. This helps plasmon absorption at a longer wavelength due to the collective plasmon oscillation of the aggregated system [48].

**Figure 11:**
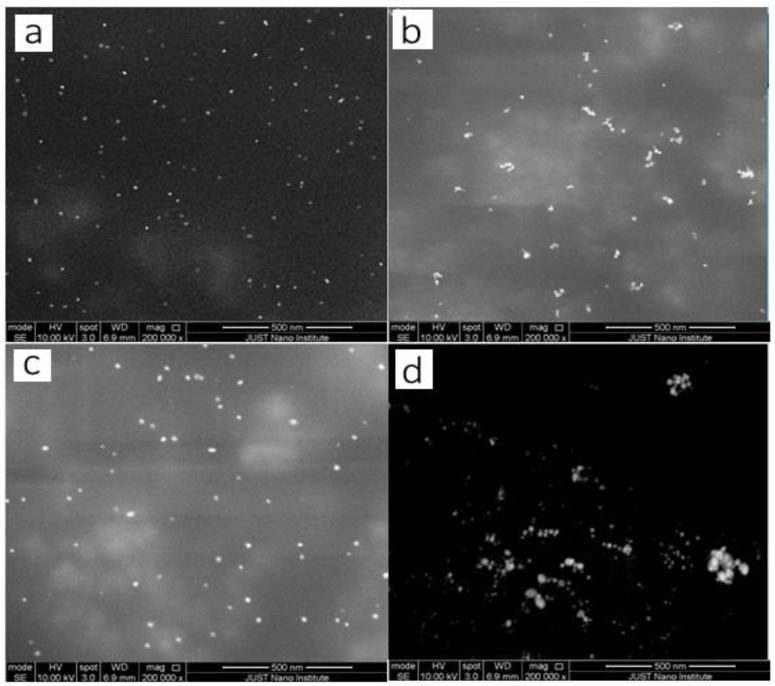
SEM images of the GNPs produced under different conditions. (**a**) GNPs made in a microreactor with a concentration of HAuCl_4_ of 0.7 mM and in the presence of PVA (after 900 s). (**b**) GNPs produced in a microreactor with a concentration of HAuCl_4_ of 0.7 mM in the presence of PVA (after one year). (**c**) GNPs prepared with a conventional method with 0.7 mM HAuCl_4_ in the presence of PVA (after 900 s). (**d**) GNPs created using the conventional method with a HAuCl_4_ concentration of 0.7 mM in the absence of PVA (after one year).

The Microreactor wall plays a crucial role in obtaining ultra-small particles. Figure 11 displays SEM images of the prepared gold nanoparticles. (Figure 11a–d) display the shape and morphology of nanoparticle size obtained as a function of concentrations of gold precursor. The diameter of the GNPs obtained when the concentration of HAuCl_4_ is 0.7 mM was 11.9 nm. This is the size range for batch-produced gold nanoparticles made using the Turkevich method. The outcome shows that without interaction between the precursor and the wall, there was no enhancement of nucleation. The continuous flow microfluidic system has good mixing and short residence time distribution to achieve low monodispersity of particle size. Figure 11b depicts the SEM-scanned image. The particles vary in shape and size, and they are slightly agglomerated. The average particle size ranged from 20 to 25 nm. Figure 11, c shows the prepared GNPs in the batch method. It was noted that the size of GNPs was larger than prepared GNPs using a microreactor. This is indicated good contact between the wall and reagents. The diameter of the GNPs obtained in this method was 25.9 nm. It was noticed that NP in microreactor have much less agglomeration. They are almost monodispersed which very good.

The effect of PVA as a stabilizer on the size of GNPs was investigated. The gold nanoparticles are of different shapes and sizes, and they are also agglomerated in the absence of PVA, as shown in Figure 11d. The mean particle size was 37 nm. It was also observed that no clumps formed in the presence of PVA. This demonstrates the potency of PVA as a stabilizing agent in preventing growth and drastically reducing the size of GNPs. The absence of the stabilizer (PVA) increased the size of GNPs over time, as shown in Figure 11d. These findings are consistent with those of Nur et al. [31].

### 2.3. The Effect of Temperature

**Figure 12:**
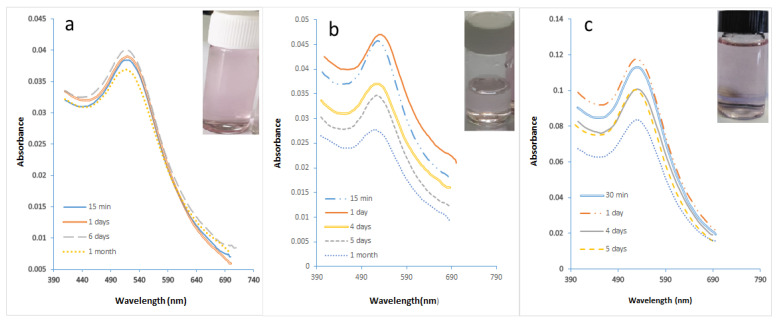
The ultraviolet (UV) spectra of GNP samples made in a microreactor at various temperatures. (**a**) at 291.15 K. (**b**) at 328.15 K. (**c**) The UV spectra for samples of GNPs that were conventionally prepared at 328.15 K. (Cons of SC, SB, and HAuCl_4_ = 0.1 mM, total SB and gold flowrate with SC = 1.66 × 10^−5^ L/s, volumetric ratio of SB/HAuCl_4_ = 4:10, pH_i_ = 3).

**Figure 13:**
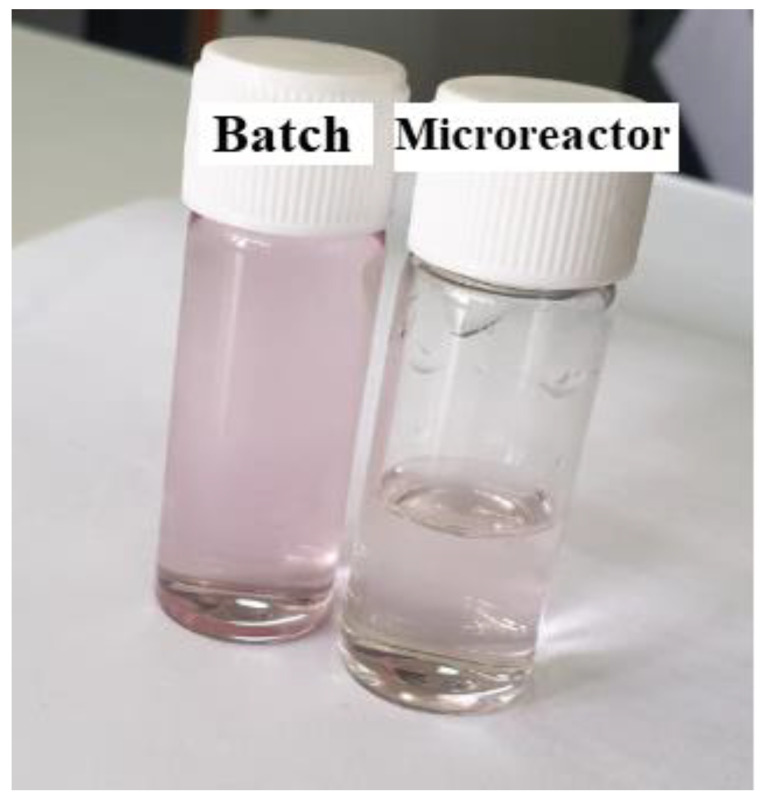
Colors for GNPs samples prepared in a microreactor at 328.15 K. (Cons of SC, SB, and HAuCl_4_ = 0.1 mM, total flowrate of SB and gold with SC = 1.66 × 10^−5^ L/s, volumetric ratio of SB/HAuCl_4_ = 4:10, pH_i_ = 3).

The UV spectra of GNPs prepared at various temperatures are shown in Figure 12a–c. At a temperature of 291.15 K, large-sized GNPs (14.44 nm) were produced. The size of the GNPs was decreased to 9.58 nm by raising the temperature to 328.15 K. The particle size was 12.99 nm when the temperature reached 308.15 K. This is in agreement with the results of the first section of this study, which found that a temperature of 308.15 K was ideal for producing small, uniform GNPs. It was noted that the microreactor’s high surface area/volume ratio, high mass transfer, and high heat transfer all contribute to its high efficiency in producing smaller GNPs. The absorbance curve width increased with decreasing temperature, as shown in Figure 12b. Depending on the phenomenon of Full Width at Half Maximum (FWHM), it is explained that reducing temperature leads to the increasing size of GNPs [49]. It is evident that the production of smaller-sized GNPs is a result of rising temperatures. These findings align with those of Wuithschick et al. [50]. The reduction in particle size is also evident from the color difference as shown in Figure 13. 

The thermal oxidation of TSC is greatly influenced by temperature. At higher temperature, more dicarboxy acetone (DCA) is produced than it does at lower temperatures. Higher temperatures cause fast nucleation sites to form, which results in a large number of small-sized nanoparticles being created. More nucleation sites are created at higher temperatures because of the reduction of the nucleation activation energy. Additionally, the consumption of gold ions at higher temperatures is accelerated by a direct correlation between reaction rate and reaction heat, which is the main cause of the formation of more nuclei and increased kinetic energy, which in turn leads to an increase in the frequency of GNP collisions. This generates small GNPs at high temperatures. The best temperature for producing small-scale GNPs with good stability was found to be 308.15 K.

**Figure 14:**
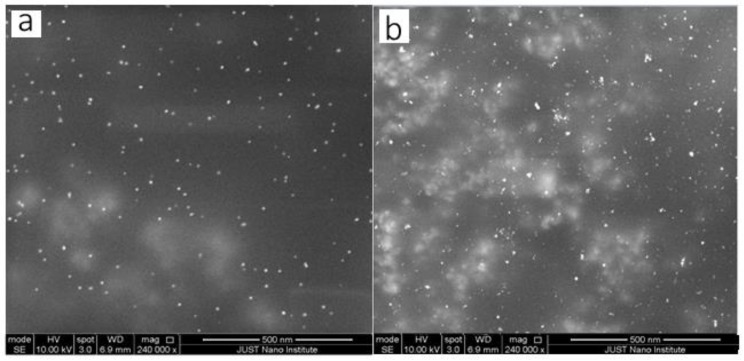
SEM images of the GNPs prepared at different temperature. (**a**) at high temperature (328.15 K), (**b**) at room temperature (291.15 K).

**Figure 15:**
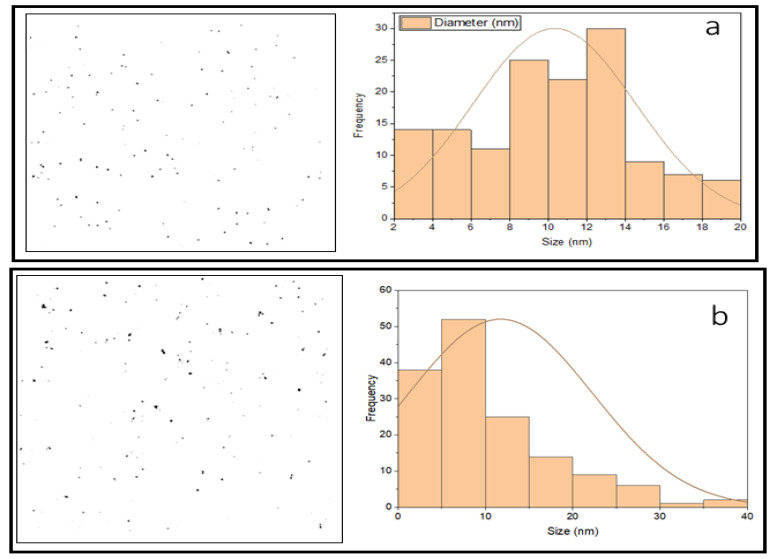
SEM images and histogram of percent frequency distribution of the GNPs prepared at different temperatures. (**a**) at 328.15 K, (**b**) at 291.15 K.

The effect temperature has on the gold nanoparticle size was investigated by SEM images, as shown in Figure 14 and Figure 15. First, as the temperature rises, more nuclei are formed because the rate of gold ion reduction and seed particle formation increases. Second, raising the temperature encourages the equilibrium of the gold precursor to shift to more hydroxylated species of HAuCl4, which have lower reactivity and produce fewer nuclei. As illustrated in Figure 6, the size and size distribution decrease as temperature rises. At 328.15 K, the largest particle size obtained was 11 nm, which is still a small size in comparison to the typical particle size in the Turkevich method. This implies that the increased nucleation rate by the tubing wall has the greatest influence on particle size, and that the effect of decreasing reactivity of hydroxylated gold precursor species has no effect on this synthesis. Furthermore, a higher temperature accelerates the reduction rate, which improves the nucleation rate. As a result, the greater the number of gold atoms available during the nucleation stage, the more seed particles are produced, and the total precursor is distributed to a greater number of particles, resulting in smaller and gold nanoparticles at higher temperatures.

### 2.4. The Effect of Flowrate

**Figure 16:**
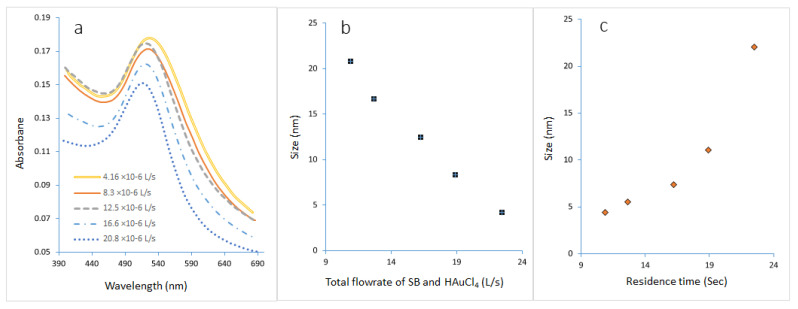
(**a**) The UV-vis spectra of GNP samples prepared in a microreactor at various flow rates of (SB and HAuCl_4_). (**b**) The impact of flow rate on the size of the final particle. (**c**) The impact of residence time on particle size. (Cons of SC, SB, and HAuCl_4_ = 0.1 mM, T = 308.15 K, volumetric ratio of SB/AuCl_4_ = 4:10, and pH = 3).

Figure 16a shows the UV-spectra of GNPs prepared at different flow rates for both gold precursors mixed with sodium citrate and sodium borohydride. The final particle size of the GNPs depended on the residence time. It can be seen that when the flow rate increased from (4.16 to 20.8) × 10^−6^ L/s, the peak of maximum wavelength shifted from right to left, as seen in Figure 16b. The UV spectrum’s sharp peak appeared at a flow rate of 20.8 × 10^−6^ L/s. These results indicated that the flow rate of 20.8 × 10^−6^ L/s has the smallest size from GNPs, which is the highest population of small clusters. Also, it was noted that monodisperse clusters could be obtained by microfluidic mixing at high flow rates. Moreover, the dark fouling was quite easily removed by increasing the flow rate, while the red and blue fouling could only be removed by aqua regia. It was noted that potent bubble figuration due to the decomposition of NaBH_4_ in the microreactor assists in breaking the structure of the reactant into smaller bits and in accelerating the mixing process. Figure 16c presents the relationship between residence time and the size of the resulting nanoparticles. The size of GNPs was confirmed to decrease as a function of increasing flow rate (decreasing residence time).

## 3. Materials and Methods

The experimental setup, materials used, the various techniques for the synthesis of GNPs, the effect of the various parameters on the synthesized GNP using the proposed method in the paper, and the various characteristics performed on the synthesized GNP are all described in this section.

### 3.1. General Note

All solutions were prepared with ultrapure water (pH = 5.5) and stored out of sunlight. Gold chloride is dissolved in water and ethanol and decomposes when temperatures exceed 433 K and in sunlight. It is also considered a dangerous solution. Mixtures were prepared under the fume hood because fumes appear, including Cl_2_, NO, NO_2_ and aqua regia, which is a mixture of two concentrated acids (chloric acid and nitric acid). Therefore it is used with great caution.

### 3.2. Chemicals

The materials used in the study are gold sheets made of 99.99% gold. Sodium citrate tribasic dehydrate with a purity of 99.9%, obtained from Fischer Chemical, Guangzhou, China. Sodium borohydride with a purity of 99.9% and NaOH were purchased from Sigma-Aldrich, Darmstadt, Germany. HCl and HNO_3_ were obtained from Biosolve Chimie, Dieuze, France. PVA is purchased from α-alpha chemika, Maharashtra, India. ultra-pure water, and aqua regia (HCl/HNO_3_, 3: 1 *v*/*v*).

### 3.3. Experimental Setup in Microreactor Mode

**Figure 17:**
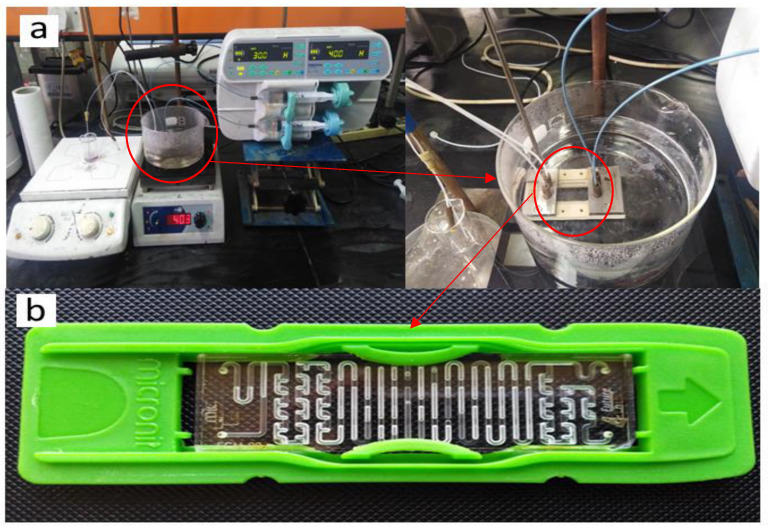
(**a**) An experimental setup for producing GNPs in a microreactor (**b**) A borosilicate glass microreactor with two inlets and two outlets.

The reactor used in this experiment (M-121 Basic Quench) is made of borosilicate glass with a total volume of 92 µL. The outer dimensions of the wafer are 15.3 × 2.2 × 45.3 mm, while the channel width is 600 µm, the channel depth is 500 µm, the bottom diameter hole is 1170 µm, and the top diameter hole is 300 µm. The reactor is fixed inside a plastic holder, as shown in Figure 17b. The reactor has two inlets and two outlets that can be connected with capillary tubes. The reacting solutions are loaded into 0.01 L plastic syringes that are fixed on a digitally controlled dual syringe pump that is used to establish a continuous feed to the reactor. The temperature inside the reactor is controlled by immersing the reactor in a hot oil bath with a controlled temperature setting. The samples were collected in a beaker containing the stabilizer (PVA) at the outlet of the microreactor. The beaker was placed on the stirrer to achieve the homogenous mixing of the resulting solution, as shown in Figure 17a.

### 3.4. Synthesis of GNPs in Batch Mode

In this step, GNPs were synthesized using the modified Martin method. In brief, 0.01 L of different concentrations of SC (0.1, 0.5, and 0.9 mM) were mixed with 0.01 L of different concentrations of HAuCl_4_ (0.02, 0.1, and 0.3 mM) for 1800 s with vigorous stirring from 300 to 700 rpm, followed by 1–10 × 10^−3^ L of NaBH_4_ (0.1, 0.3, and 0.7 mM). This experiment was done at various temperatures ranging from 298.15 to 393.15 K. The pH of the gold solution was initially adjusted from 2 to 8 by adding hydrochloric acid (HCl) or sodium hydroxide (NaOH).

### 3.5. Synthesis of GNPs in Microreactor Mode

First, a 3.76 mM gold-stock solution was prepared by dissolving 87 × 10^−6^ kg of gold pure in 0.018 L of HCl and 0.001 L of nitric acid at room temperature until it became a homogeneous mixture. This mixture was left on the hot plate to remove all gases from the mixture (such as NO_2_ or NO), where all excess nitric acid evaporated from the mixture when the reddish-brown fumes stopped, as shown in reaction 1. In this step, rock wool was used to maintain the required temperature for the solution. This step was repeated more than once to reach the required pH value of 2. The obtained solution was made up to 0.1 L, and then it was analyzed using atomic absorption spectroscopy. Next, at room temperature, 1 × 10^−3^ kg of SC was dissolved in 0.1 L of ultra-pure water to make a 34 mM solution. At room temperature, 0.189 × 10^−3^ kg of NaBH_4_ was then dissolved in 0.05 L of ultra-pure water to form a 100 mM solution. This solution was prepared fresh each time. The experimental conditions used for the synthesis of GNPs using the microreactor technique are chosen based on the optimal results obtained from the batch synthesis experiments. These conditions are: HAuCl_4_, SC, and SB concentrations, the volumetric ratio of NaBH_4_/HAuCl_4_, the initial pH, temperature, and mixing rate. A mixture of HAuCl_4_ of 0.1 mM and SC of 0.1 mM was prepared and loaded into a 0.01 L plastic syringe. The solution is then fed to the inlet of the reactor at a flow rate of 5.55 × 10^−6^ L/s After a stable flow inside the reactor has been established, a freshly prepared solution of 0.1 mM SB is fed to the second inlet of the reactor at a flow rate of 1.11 × 10^−5^ L/s. The total residence time inside the reactor was 5.5 s.

(1) $$\mathrm{Au}+4\mathrm{HCL}+{\mathrm{HNO}}_{3}\to {\mathrm{AuHCl}}_{4}+2H_{2}O+\mathrm{NO}$$

### 3.6. Characterization of Gold Nanoparticles

The stock gold concentration was measured using the Unicam Atomic Absorption Spectrometer Model (SOLAAR M5) made by Unicam Atomic Absorption, UK. From a standard gold solution with a concentration of 1000 ppm, three samples with a value of 1, 5, and 10 ppm were prepared. The same concentrations were then prepared from the gold solution that was prepared in the laboratory. The results were taken to an atomic absorption spectroscopy device to find out the concentration of the prepared gold through the amount of their absorption of the exposed radiation, which exposes the electrons to tremendous energy that stimulates them to move to higher energy levels. Light is absorbed at a specific wavelength, and each metal differs from the other in its wavelength. The wavelength of the pure gold element was determined on the device at 530 nm [51]. The solution was diluted to 0.01 L with ultra-pure water. Absorption measurements were performed using a UV-Vis Spectrophotometer Model (DR 5000) made in the USA, in the wavelength range of (400–700) nm using (10 × 10) mm quartz cuvettes. The size and size distribution of GNPs were analyzed by a Dynamic Light Scattering (DLS) device model (Malvern) manufactured in the United Kingdom. Samples were kept in cuvettes made from polystyrol or polystyrene (10 × 10 × 45) mm. All samples were diluted with 1% ultra-pure water before analysis. 

## 4. Conclusions

This work shows that using microfluidic methods helps better control the reaction conditions for producing (10–11) nm of GNPs in the presence of the PVA. The sizes of GNPs in the absence of PVA ranged from 11.76 to 13.54 nm, while the sizes of GNPs prepared using the batch method ranged from 17.37 to 26.761 nm. This confirmed that the size of GNPs resulting from using the microreactor is smaller than those produced using the batch reactors and that the temperature control for the reaction is much easier. Also, it was observed that the best size of GNPs for remaining stable for the longest possible time is at concentration levels lower than 2 mM. In the future, studying the effect of the volume of the stabilizing agent added to gold precursors on the size of GNPs is recommended. Studying more than one stabilizing agent and comparing them in the size of the resulting GNPs is also recommended. It was noticed that NPs made in the microreactor have better spherical morphology than the NP made in batch. Also, it can be seen that GNP in microreactor have much less agglomeration. 

## Data Availability

The data presented in this study are available on request from the corresponding author.

## Sample Availability

Samples of the compounds are not available from the authors.
